# Case report: *Nocardia gipuzkoensis* infection in an immunocompetent patient diagnosed by metagenomic next-generation sequencing and whole genome sequencing

**DOI:** 10.3389/fimmu.2022.1053914

**Published:** 2022-12-09

**Authors:** Chengxin Liu, Juhua Yang, Huiting Huang, Shaofeng Zhan, Xintian Xia

**Affiliations:** ^1^ The First Clinical Medical School, Guangzhou University of Chinese Medicine, Guangzhou, China; ^2^ The First Affiliated Hospital, Guangzhou University of Chinese Medicine, Guangzhou, China; ^3^ Vision Medicals Co., Ltd., Guangzhou, China

**Keywords:** nocardiosis, antibiotic susceptibility, pathogen identification, metagenomic next-generation sequencing, whole genome sequencing, case report

## Abstract

The infection of *Nocardia gipuzkoensis* is a relatively uncommon form of pulmonary nocardiosis seen in clinical patients. In general, nocardiosis tends to occur in patients with immune deficiency. Here, we report a 23-year-old female who was admitted to the hospital due to cough and sputum production over 10 years, diagnosed with bronchiectasis. The *N. gipuzkoensis* infection was identified by metagenomic next-generation sequencing and whole genome sequencing. Imipenem/cilastatin and compound sulfamethoxazole tablets were used to control the infection and the pulmonary inflammation subsided gradually.

## Highlights

- *N. gipuzkoensis* can be responsible for opportunistic infections in immunocompetent humans.
- The mNGS combined with WGS is reliable for identifying and typing strains of species.
- Identification and elimination of *Nocardia* might be the way for preventing bronchiectasis caused due to recurrent infections.

## 1 Introduction

*Nocardia* is a genus of rod-shaped bacteria with the characteristics of weakly staining Gram-positive and catalase-positive. It contains a total of approximately 134 species according to Wikipedia (last revised on August 24, 2022), and an increasing number of species have been recognized as human pathogens. The common clinical presentations contain pulmonary and cutaneous nocardiosis, which are acquired by inhalation of the bacteria or through traumatic introduction (1, 2). *Nocardia* is considered a major opportunistic pathogen, especially affecting patients with impaired immune systems. However, *Nocardia* infections in immunocompetent patients have been increasingly reported (3, 4). In immunocompetent patients, chronic obstructive pulmonary disease and bronchiectasis could be considered to represent risk factors for pulmonary *Nocardia (* 5–7). It is worth mentioning that a novel drug-resistant community-acquired *Nocardia spp* was identified in a patient with bronchiectasis (8).

Metagenomic NGS (mNGS) is an incredibly sensitive detection method to understand which microbes are present and in what proportions *via* running all nucleic acids in a sample to the reference genomes (9, 10). Compared with traditional culture methods, mNGS provides a faster detection turnaround time and identifies theoretically all infectious pathogens in clinical specimens by sequencing DNA or RNA fragments; the detection rate would not be reduced while the patients had received antibiotic treatment (11). On the other hand, cultivating cannot classify specific species; however, mNGS was more efficient and sensitive in detecting different *Nocardia* (12). Its possible clinical applications are tremendous, especially in the discovery of pathogens from respiratory samples, blood, and other body fluid.

Whole genome sequencing (WGS) is a testing method that determines the order of nucleotide bases in the genome of an organism, therefore assessing the bacterial genomic features and possible origins. WGS procedures have qualified significant contributions in virology areas, especially for the rapid identification and classification of pathogens (13). So far, WGS has been used for revealing the genetic diversity, taxonomic structure, and the evolution relationship of the *Nocardia (* 14).

In this report, we present a case of an immunocompetent patient suffering from recurrent lung infections caused by *Nocardia gipuzkoensis*, which was identified through the mNGS combined with WGS; its diagnosis and treatment processes were reported as well.

## 2 Case presentation

**Figure 1:**
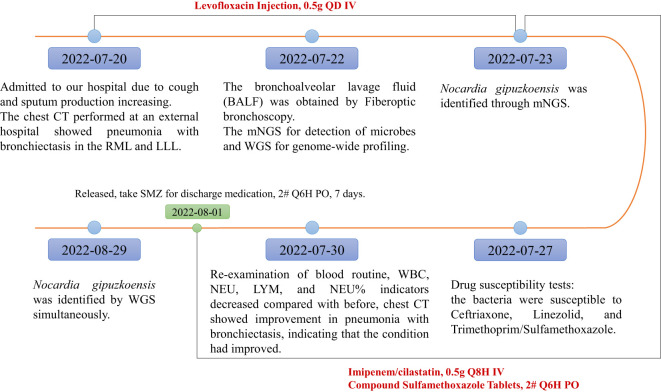
The timeline during hospitalization.

A 23-year-old female was admitted to the Respiratory Department of The First Affiliated Hospital, Guangzhou University of Chinese Medicine, with recurrent cough and phlegm that had been present for more than 10 years. The timeline during hospitalization is shown in **Figure 1**, and the complete case progress record in detail is reported as follows.

The patient was diagnosed with bronchiectasis on May 29, 2021, according to the symptoms of chronic cough and sputum production as well as chest computed tomography (CT), which demonstrated pneumonia with bronchiectasis in the right middle lobe (RML) and left lower lobe (LLL) principally. The patient had not received further diagnosis and treatment systematically. By asking for medical history and consulting the patient’s medical record, we noticed the patient had been hospitalized for the same illness on February 16, 2022. The IgM test of respiratory tract pathogens showed that Legionella pneumophila and mycoplasma pneumoniae were positive. The patient got better and was discharged after being given antibiotics containing Nemonoxacin, Cefoperazone-sulbactam, and Piperacillin-sulbactam. However, the patient did not undergo fiberoptic bronchoscopy to obtain bronchoalveolar lavage fluid (BALF) for mNGS but only identified the potential infectious pathogens by the IgM test.

Approximately 10 days before this admission, the patient developed an aggravated cough with sputum production and even a little hemoptysis, so the patient was admitted to the respiratory department for treatment on July 20, 2022. No obvious abnormalities were observed in vital signs. Chest CT performed at an external hospital on June 26, 2022, showed more serious pneumonia with bronchiectasis in the RML and LLL. The laboratory investigations after hospital admission prompted normal C-reactive protein, blood sedimentation rate, and normal kidney and liver function. However, blood analysis on the day of admission showed that the total white blood cells (WBC) were 9.44*10^9/L, the total number of neutrophils (NEU) was 6.79*10^9/L, the total lymphocyte count (LYM) was 2.03*10^9/L, and neutrophil percentage (NEU%) was 71.9%. Levofloxacin injection was the initial empiric antimicrobial therapy administered.

**Figure 2:**
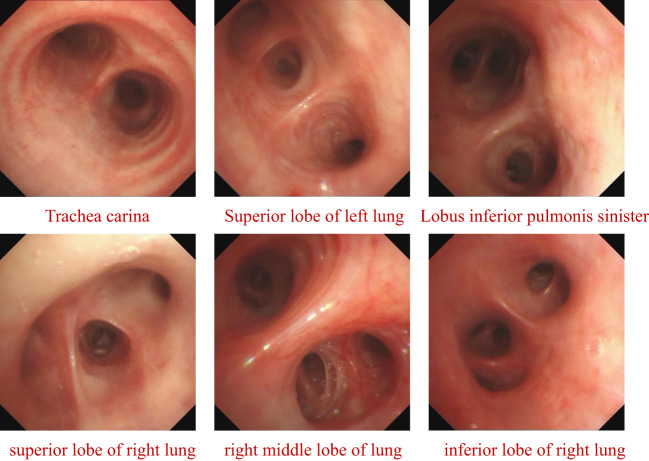
The lesion of Bronchus on fiberoptic bronchoscopy.

To identify the pathogen as soon as possible, fiberoptic bronchoscopy was performed on hospital day 2 to obtain BALF for mNGS. Airway secretions can be found *via* fiberoptic bronchoscopy; the lesions of the bronchus are presented in **Figure 2**. The *N. gipuzkoensis* infection was identified by mNGS (results of this analysis are shown in **Table 1**). The antibiotic regimen was then adjusted to Imipenem/cilastatin, 0.5g Q8H IV, and compound sulfamethoxazole tablets (SMZ), 2# Q6H PO (composed of Trimethoprim 80 mg and sulfamethoxazole 0.4 g per tablet). WGS technology was arranged for genome-wide profiling.

**Table 1:** Identification of *Nocardia* in BALF Samples using metagenomic next generation sequencing.

Genus				Species		
Gram’s staining	Genus Name	Relative abundance	Sequences	Species Name	Confidence level	Sequences
G+	*Nocardia*	2.9%	2,887	*N. gipuzkoensis*	99%	239

**Figure 3:**
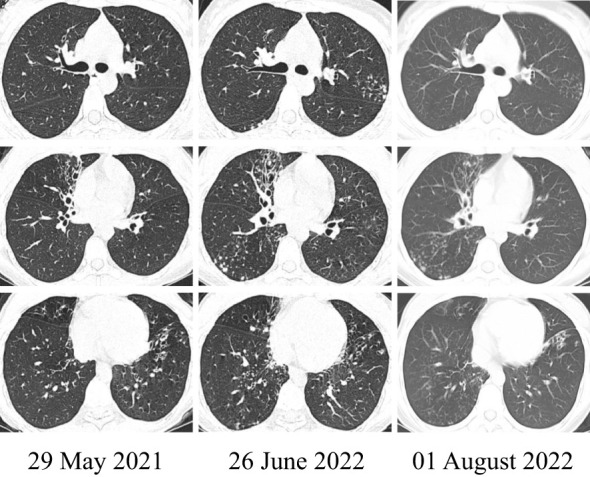
The representative image: changing in the patient’s chest CT. The CT on June 26, 2022, compared with May 29, 2021, showed more serious pneumonia with bronchiectasis in the RML and LLL. However, after the identification of *Nocardia* species and systemic treatment, the imaging on August 1, 2022, showed improvement.

On hospital day 7, drug susceptibility tests according to the standards of U.S. Clinical and Laboratory Standards Institute (CLSI) (15) demonstrated sensitivity to Ceftriaxone, Linezolid, and Trimethoprim/Sulfamethoxazole (the antibiotic susceptibility of *Nocardia* from the BALF is shown in **Table 2**). Therefore, this patient achieved unchanged antibiotic treatment. On July 30 (day 10 after admission), the repeat blood routine examination results, WBC, NEU, LYM, and NEU% were 4.93*10^9/L, 2.60*10^9/L, 1.68*10^9/L, and 52.7%, respectively, decreased compared with before, indicating that the condition had improved. A chest CT scan demonstrated that bronchiectasis was evident in the RML and LLL with multiple nodular, flocculent shadow, and tree-in-bud signs. Imaging changes over time showed progressive improvement in pneumonia with bronchiectasis (**Figure 3**). Finally, she was discharged on August 1, 2022, and 7 days of SMZ treatment was continued.

**Table 2:** The antibiotic susceptibility of *Nocardia* from the bronchoalveolar lavage fluid.

Num.	Antibiotics	MIC for category(μg/mL)			MIC for *Nocardia*	Interpretation
		Susceptible	Intermediate	Resistant		
1	Ceftriaxone	≤8	16-32	≥64	0.25	S
2	Linezolid	≤8	–	–	0.50	S
3	Trimethoprim/Sulfamethoxazole	≤2/38	–	≥4/76	0.094	S
4	Meropenem	–	–	–	0.19	–

MIC, Minimum inhibitory concentration, is tested by using agar dilution via E test measured, based on the CLSI standards. S, Susceptible.

**Figure 4:**
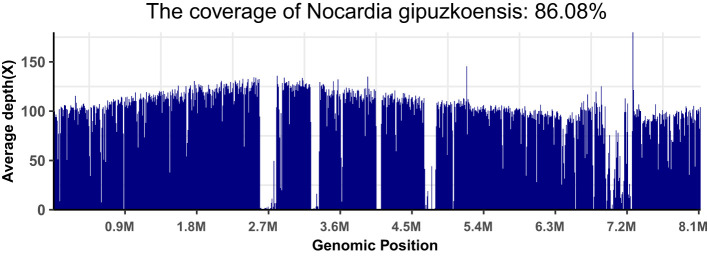
The coverage map of *N. gipuzkoensis*.

**Figure 5:**
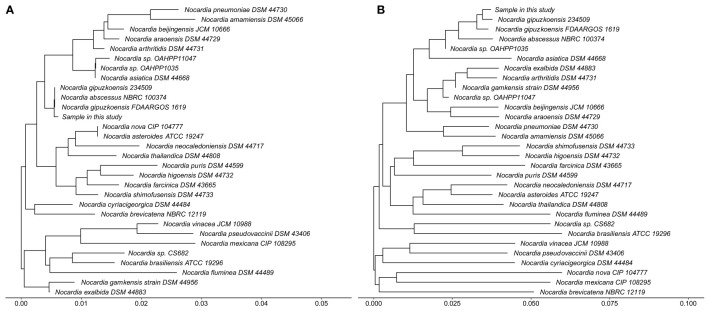
Phylogenetic trees based on the sequences of 16S rRNA gene **(A)** and housekeeping genes **(B)** show the phylogenetic position of the sample in this study within the evolutionary radiation of the genus *Nocardia*. The housekeeping genes contains *hsp65*, *secA1*, *gyrB*, and *rpoB*. The methods of determining phylogeny are following the steps: comparing multiple sequences by MAFFT, extracting conserved sequences by Gblocks, and then constructing the Neighbor-Joining by TreeBeST.

After waiting approximately a month, we received the patient’s WGS results and identified the infection of *N. gipuzkoensis*. The coverage map of *N. gipuzkoensis* is shown in **Figure 4**. The sequences identified by the whole genome were 54,536. The phylogenetic trees were constructed based on the sequences of 16S rRNA gene and housekeeping genes and are shown as **Figure 5**.

At the same time, the strain was validated by quantitative PCR (qPCR). The primers for *N. gipuzkoensis* used in qPCR detection were Forward, 5’- GGCACGACCGATAACCGAACG-3’, and Reverse, 5’-ATCCATCCGCTACCGCTCCTTC-3’. We designed the primers based on the *N. gipuzkoensis* sequences from the Basic Local Alignment Search Tool. We tested their amplification specificity with Primer-BLAST software. No target templates were found in the Refseq mRNA database (organism limited to homo sapiens). Using our qPCR assay, the BALF sample of three replicates was positive compared with the sample of another healthy adult.

## 3 Discussion

Nocardiosis is an uncommon and opportunistic bacterial infection caused by inhalation or direct inoculation. This patient lived in an environment where mud for turtles was piled up at the bottom of the bed for a long time, and this is considered to be an important cause of *Nocardia* infection. It is especially interesting that *N. gipuzkoensis* was finally identified through mNGS combined with WGS.

*N. gipuzkoensis* was initially isolated from the sputum of an old female patient, who was diagnosed with bronchiectasis before, and expectoration was the main symptom (16). This seems to indicate that elderly patients with chronic lung diseases are more susceptible to *N. gipuzkoensis*, but no more cases have been reported.

The use of corticoid treatment in people with chronic lung disease, such as bronchiectasis, was a risk factor for pulmonary nocardiosis (7). A rare case of pulmonary nocardiosis has also been reported in a non-immunocompromised patient who had bronchiectasis without corticoid treatment (17). Lung consolidation, nodules, pleural involvement, and chest wall extension were the frequent CT findings of pulmonary nocardiosis (18). In this patient, the presentation of bronchiectasis with pneumonia progressed over the past year. The patient had recurrently suffered lower respiratory tract infections since the diagnosis of bronchiectasis in 2021 but improved with the treatment of SMZ after the identification of *Nocardia*. Before the nocardiosis diagnosis, the CT in 2022 compared with 2021 showed more serious pneumonia with bronchiectasis in the RML and LLL. However, after the identification of *Nocardia* and receiving systemic treatment, the next imaging showed an improvement. We speculated that infection of the *Nocardia* might be a significant etiology causing bronchiectasis. The elimination of *Nocardia* could be the way for preventing bronchiectasis caused due to recurrent infections. In the case of bronchiectasis with recurring pneumonia, BALF collected *via* fiberoptic bronchoscopy should be performed as soon as possible, and the pathogenic bacteria should be identified by high-throughput sequencing technology. The using of mNGS combined with WGS is reliable and practical for identifying and typing strains of species.

## Limitation

We have reported a case of an immunocompetent patient suffering from recurrent lung infections caused by the infection of *N. gipuzkoensis*. Nevertheless, we did no laboratory studies to evaluate these bacterial attributes in an attempt to clarify the pathogenesis of the disease. Next, an animal experiment could be considered to test the pathogenicity *in vivo*.

## Data availability statement

The original contributions presented in the study are included in the article/supplementary materials. Further inquiries can be directed to the corresponding authors.

## Ethics statement

Written informed consent was provided by the patient to allow the case details and images to be published. Our report was approved by ethics committee of The First Affiliated Hospital, Guangzhou University of Chinese Medicine (K-2022-108).

## Author contributions

Article design and writing: CL. The collection and detection of clinical sample: JY. Patient management and treatment: HH, SZ and XX. All authors contributed to the article and approved the submitted version.

## References

[1] Ott SR Meier N Kolditz M Bauer TT Rohde G Presterl E . Pulmonary nocardiosis in Western Europe-clinical evaluation of 43 patients and population-based estimates of hospitalization rates. Int J Infect Dis (2019) 81:140–8. doi: 10.1016/j.ijid.2018.12.010 30658169

[2] Laube H . Primary cutaneous nocardiosis. Deutsches Arzteblatt Int (2019) 116:362. doi: 10.3238/arztebl.2019.0362a PMC6637659 31288918

[3] Abe S Tanabe Y Ota T Fujimori F Youkou A Makino M . Case report: pulmonary nocardiosis caused by nocardia exalbida in an immunocompetent patient. BMC Infect Dis (2021) 21:776. doi: 10.1186/s12879-021-06416-w 34372796 PMC8351411

[4] Wintheiser GA Venable ER Temesgen Z . Disseminated nocardia in an immunocompetent host. Mayo Clinic Proc (2021) 96:847–8. doi: 10.1016/j.mayocp.2020.11.019 33814090

[5] Menéndez R Cordero PJ Santos M Gobernado M Marco V . Pulmonary infection with nocardia species: a report of 10 cases and review. Eur Respir J (1997) 10:1542–6. doi: 10.1183/09031936.97.10071542 9230244

[6] Garcia-Bellmunt L Sibila O Solanes I Sanchez-Reus F Plaza V . Pulmonary nocardiosis in patients with COPD: characteristics and prognostic factors. Archivos bronconeumol (2012) 48:280–5. doi: 10.1016/j.arbr.2012.06.006 22656187

[7] Ercibengoa M Càmara J Tubau F García-Somoza D Galar A Martín-Rabadán P . A multicentre analysis of nocardia pneumonia in Spain: 2010-2016. Int J Infect Dis (2020) 90:161–6. doi: 10.1016/j.ijid.2019.10.032 31693939

[8] Li Z Li Y Li S Li Z Mai Y Cheng J . Identification of a novel drug-resistant community-acquired nocardia spp. in a patient with bronchiectasis. Emerging Microbes infections (2022) 11:1346–55. doi: 10.1080/22221751.2022.2069514 PMC9132467 35450515

[9] Bragg L Tyson GW . Metagenomics using next-generation sequencing. Methods Mol Biol (Clifton N.J.) (2014) 1096:183–201. doi: 10.1007/978-1-62703-712-9_15 24515370

[10] Miller S Chiu C . The role of metagenomics and next-generation sequencing in infectious disease diagnosis. Clin Chem (2021) 68:115–24. doi: 10.1093/clinchem/hvab173 34969106

[11] Miao Q Ma Y Wang Q Pan J Zhang Y Jin W . Microbiological diagnostic performance of metagenomic next-generation sequencing when applied to clinical practice. Clin Infect Dis (2018) 67:S231–s240. doi: 10.1093/cid/ciy693 30423048

[12] Weng SS Zhang HY Ai JW Gao Y Liu YY Xu B . Rapid detection of nocardia by next-generation sequencing. Front Cell infection Microbiol (2020) 10:13. doi: 10.3389/fcimb.2020.00013 PMC7040243 32133300

[13] Quiñones-Mateu ME Avila S Reyes-Teran G Martinez MA . Deep sequencing: becoming a critical tool in clinical virology. J Clin Virol (2014) 61:9–19. doi: 10.1016/j.jcv.2014.06.013 24998424 PMC4119849

[14] Xu S Li Z Huang Y Han L Che Y Hou X . Whole genome sequencing reveals the genomic diversity, taxonomic classification, and evolutionary relationships of the genus nocardia. PloS Negl Trop Dis (2021) 15:e0009665. doi: 10.1371/journal.pntd.0009665 34437546 PMC8437295

[15] Woods GL Brown-Elliott BA Conville PS Desmond EP Hall GS Lin G . CLSI standards: Guidelines for health care excellence, susceptibility testing of mycobacteria, nocardiae, and other aerobic actinomycetes. Wayne (PA: Clinical and Laboratory Standards Institute Copyright (2011). 31339680

[16] Nouioui I Cortés-Albayay C Neumann-Schaal M Vicente D Cilla G Klenk HP . Genomic virulence features of two novel species nocardia barduliensis sp. nov. and nocardia gipuzkoensis sp. nov., isolated from patients with chronic pulmonary diseases. Microorganisms (2020) 8:1517. doi: 10.3390/microorganisms8101517 33019781 PMC7600791

[17] Cremades MJ Menéndez R Santos M Gobernado M . Repeated pulmonary infection by nocardia asteroides complex in a patient with bronchiectasis. Respiration Int Rev Thorac Dis (1998) 65:211–3. doi: 10.1159/000029264 9670306

[18] Kanne JP Yandow DR Mohammed TL Meyer CA . CT findings of pulmonary nocardiosis. AJR Am J roentgenol (2011) 197:W266–72. doi: 10.2214/AJR.10.6208 21785052

